# Predominance of *bla*_NDM_- and *bla*_IMP_-Harboring *Escherichia coli* Belonging to Clonal Complexes 131 and 23 in a Major University Hospital

**DOI:** 10.3390/medicina60091528

**Published:** 2024-09-19

**Authors:** Muhammad Shafiq, Iftikhar Ahmed, Muhammad Saeed, Abdul Malik, Sabiha Fatima, Suhail Akhtar, Mohsin Khurshid, Muhammad Zeeshan Hyder

**Affiliations:** 1 Department of Biosciences, COMSATS University Islamabad, Park Road, Islamabad 45550, Pakistan; mshafiquea@hotmail.com (M.S.); muhammad.saeed@comsats.edu.pk (M.S.); 2 National Culture Collection of Pakistan (NCCP), Land Resources Research Institute (LRRI), National Agriculture Research Centre, Park Road, Islamabad 45500, Pakistan; iftikhar.ahmed@parc.gov.pk; 3 Department of Pharmaceutics, College of Pharmacy, King Saud University, Riyadh 11564, Saudi Arabia; amoinuddin@ksu.edu.sa; 4 Department of Clinical Laboratory Sciences, College of Applied Medical Sciences, King Saud University, Riyadh 12371, Saudi Arabia; sabmehdi@ksu.edu.sa; 5 Department of Biochemistry, A.T. Still University of Health Sciences, Kirksville, MO 63501, USA; suhailakhtar@atsu.edu; 6 Institute of Microbiology, Government College University, Faisalabad 38000, Pakistan

**Keywords:** infection control, surveillance, MLST, carbapenems, ST131

## Abstract

*Background and Objectives*: Carbapenem resistance is a growing global challenge for healthcare, and, therefore, monitoring its prevalence and patterns is crucial for implementing targeted interventions to mitigate its impact on patient outcomes and public health. This study aimed to determine the prevalence of carbapenem resistance among *Escherichia coli* (*E. coli*) strains in the largest tertiary care hospital of the capital territory of Pakistan and to characterize the isolates for the presence of antimicrobial resistance genes. Additionally, the most prevalent sequence types were analyzed. *Materials and Methods*: A total of 15,467 clinical samples were collected from November 2020 to May 2022, underwent antimicrobial susceptibility testing, and were analyzed for antimicrobial resistance genes through conventional PCR and sequence typing using MLST. *Results*: In carbapenem-resistant *E. coli* (CR-EC), 74.19% of isolates harbored the *bla*_NDM_ gene, with *bla*_NDM-1_ (66.96%), *bla*_NDM-5_ (12.17%), and *bla*_NDM-7_ (20.87%) variants detected. Additionally, *bla*_IMP_ was found in 25.81% and *bla*_OXA-48_ in 35.48% of isolates. The presence of *bla*_CTX-M15_ and *bla*_TEM_ was identified in 83.87% and 73.55% of CR-EC isolates, respectively, while *arm*A and *rmt*B were detected in 40% and 65.16% of isolates, respectively. Colistin and tigecycline were the most effective drugs against CR-EC isolates, with both showing an MIC_50_ of 0.5 µg/mL. The MIC_90_ for colistin was 1 µg/mL, while for tigecycline, it was 2 µg/mL. MLST analysis revealed that the CR-EC isolates belonged to ST131 (24.52%), ST2279 (23.87%), ST3499 (16.13%), ST8051 (15.48%), ST8900 (9.68%), ST3329 (7.10%), ST88 (1.94%), and ST6293 (1.29%). The ST131 complex (70.97%) was the most prevalent, harboring 95.65% of the *bla*_NDM_ gene, while the ST23 complex (18.06%) harbored 62.50% of the *bla*_IMP_ gene. *Conclusions*: Implementing large-scale surveillance studies to monitor the spread of specific pathogens, along with active infection control policies, is crucial for the effective containment and prevention of future epidemics.

## 1. Introduction

Antimicrobial resistance (AMR) is a growing global concern that poses significant clinical and public health challenges. Of particular concern is the emergence of AMR, notably resistance to carbapenems in *E. coli*, which is on the rise [1,2,3]. This is significant because *E. coli* is notorious for causing both community-acquired infections (CAIs) and hospital-acquired infections (HAIs) [4]. Carbapenems are considered the mainstay for treating infections caused by multidrug-resistant (MDR) bacteria. One widespread mechanism of acquired drug resistance (ADR) to carbapenems is through the production of carbapenemases by *E. coli*. Among these carbapenemases, metallo-β-lactamases (MBLs) are particularly important. New Delhi metallo-β-lactamase (*bla*_NDM_) and imipenemase (*bla*_IMP_) are well-known MBLs worldwide, which may be co-expressed with serine-β-lactamase (*bla*_OXA-48-like_, *bla*_KPC_, etc.) [5]. Considering infection control and therapeutic management, carbapenem-resistant *E. coli* (CR-EC) are critical as they are prevalent in healthcare settings and increasingly found in the environment [6]. Furthermore, the selective pressure exerted by the widespread use of antibiotics in healthcare settings has contributed to the survival and proliferation of MBL-carrying CR-EC isolates [7].

MBL-producing bacteria, particularly those producing the NDM enzyme, are widely reported in the Indian subcontinent, Southeast Asia, and the Middle East. In Europe, the prevalence of *bla*_NDM_-producing bacteria is increasing, and cases are also being reported in the Americas, Australia, and other regions worldwide [8,9]. Studies have shown that the occurrence of *bla*_NDM_-producing bacteria is not limited to specific regions. The global spread of *bla*_NDM_ and other antimicrobial-resistant bacteria underscores the interconnectedness of healthcare systems and the need for global surveillance and collaboration to address the challenge of antimicrobial resistance. The worldwide dissemination of *bla*_NDM_-producing *E. coli* is associated with the spread of specific plasmids, such as IncF, IncA/C, and IncX, as well as other genetic elements like transposons and integrons, and certain bacterial clones [9]. These clones often exhibit high levels of resistance and have the ability to persist and spread in different regions. For example, the sequence type (ST) 131 clone of *E. coli* has been associated with the dissemination of *bla*_NDM_ and other AMR genes in various parts of the world [10]. A surveillance study covering 40 countries revealed that *bla*_NDM_ variants are responsible for 44.2% of all MBL-producing Enterobacterales [11]. Although these are extensively prevalent in the Indian subcontinent, these variants have now become endemic in the Balkan region, Northern Africa, and the Arabian Peninsula [12]. The *bla*_NDM_ variants are categorized from NDM-1 to NDM-25, with *bla*_NDM-1_, *bla*_NDM-5,_ and *bla*_NDM-7_ being frequently identified [9,13]. Additionally, the emergence of 16S rRNA methylases, which confer high-level resistance to aminoglycosides, is noteworthy. These enzymes are spreading particularly among members of Enterobacterales through plasmids. Multiple 16S rRNA methylase genes have been identified, with *arm*A and *rmt*B being widely reported in Enterobacterales [14].

*E. coli* sequence typing provides valuable information for public health surveillance, outbreak investigations, understanding pathogenesis and virulence, monitoring antimicrobial resistance, and studying the evolutionary aspects of this important bacterial species [10]. Among the widespread STs, ST131 is one of the most successful clones. ST131 acquires *bla*_CTX-M15_, conferring resistance to extended-spectrum β-lactam drugs. Now, these STs are acquiring MBLs (*bla*_NDM_, *bla*_IMP_) and other carbapenemases (*bla*_OXA-48-like_ or *bla*_KPC_), exhibiting resistance to carbapenems [10,15]. Understanding the clones associated with *bla*_NDM_-producing *E. coli* is essential for tracking transmission dynamics and implementing appropriate infection control measures.

While data on CR-EC in Pakistan are limited, a notable study from the capital city reported the molecular epidemiology of CR-EC [16]. Meanwhile, this study offers a comprehensive and updated evaluation of CR-EC prevalence and resistance gene profiles at the largest tertiary care hospital in the capital territory of Pakistan, with a substantial sample size. The aims of this study were to determine the prevalence, antimicrobial susceptibility, and molecular characteristics of CR-EC strains isolated from clinical samples at this hospital. Specifically, this study sought to assess the prevalence of CR-EC isolates carrying various carbapenemase genes, profile the presence and distribution of key antimicrobial resistance genes among CR-EC isolates, and identify and characterize the prevalent sequence types associated with CR-EC.

## 2. Materials and Methods

### 2.1. Sample Collection and Bacterial Isolation

In this study, samples were collected from November 2020 to May 2022 at the Pakistan Institute of Medical Sciences (PIMS), a government hospital in Islamabad. PIMS not only serves the capital territory but also caters patients from across Pakistan, particularly from the Khyber Pakhtunkhwa province, the upper Punjab province, and Azad Jammu and Kashmir. This study was approved by the ethical committee of Shaheed Zulfiqar Ali Bhutto Medical University, Islamabad, Pakistan (No. F.1-1/2015/ERB/SZABMU/678 dated 25 November 2020).

The demographic characteristics of patients, including age, gender, and location, were recorded. All *E. coli* isolated from various clinical samples, such as urine, pus, and blood, were included in this study. Bacterial isolation was conducted using standard microbiological practices [17]. All samples, except for urine samples, were inoculated on blood, chocolate, and MacConkey agar plates (Oxoid, Basingstoke, UK). Urine samples were inoculated on cystine–lactose–electrolyte-deficient (CLED) media using filter paper strips (MAST^®^, Bootle Merseyside, UK). The inoculated plates were incubated aerobically at 36 ± 1 °C overnight. Bacterial species were identified based on colonial morphology, Gram staining, oxidase tests, and biochemical tests using API 20E or Vitek 2 (bioMérieux, Durham, NC, USA) [17].

### 2.2. Determination of Antimicobial Susceptibility and MICs

Antimicrobial susceptibility testing (AMST) was determined using a modified Kirby–Bauer disk diffusion method with a 0.5 McFarland standard of bacterial suspension on Mueller–Hinton agar (MHA) (Oxoid, UK). A panel of antibiotic disks (Oxoid, UK) was applied, including imipenem (IMP), meropenem (MEM), doripenem (DOR), amikacin (AK), gentamicin (CN), tobramycin (TOB), ciprofloxacin (CIP), levofloxacin (LEV), co-trimoxazole (SXT), minocycline (MH), ceftriaxone (CTX), cefepime (FEP), cefoperazone/sulbactam (SCF), piperacillin/tazobactam (TZP), ampicillin (AMP), and amoxicillin/clavulanic acid (AMC). The minimum inhibitory concentrations (MICs) of all drugs including colistin (CT), polymyxin B (PB), and tigecycline (TGC) were determined using the microtiter broth dilution method for all CR-EC isolates. Zone sizes and MIC results were interpreted according to standard CLSI guidelines [18] and EUCAST guidelines where applicable [19]. *E. coli* ATCC 25922 was used as a quality control strain for AMST.

### 2.3. Phenotypic Detection of Carbapenemases and MBLs

Carbapenemase and MBL production in Enterobacterales was determined using the modified carbapenem inactivation method (mCIM) and EDTA-CIM (eCIM), respectively, as described by CLSI [18]. While mCIM results can be interpreted independently, eCIM results must be interpreted in conjunction with mCIM results.

Briefly, a 1 µL loopful of bacteria from an overnight blood agar plate was incubated in 2 mL of tryptic soy broth (TSB) and vortexed briefly. A meropenem (10 µg) disk was then immersed in the suspension and incubated aerobically at 35 ± 2 °C for 4 h ± 15 min. Meanwhile, a 0.5 McFarland suspension of *E. coli* ATCC 25922 in normal saline was prepared just before the completion of the TSB–meropenem disk suspension incubation. This suspension was then inoculated on Mueller–Hinton agar (MHA) plates, which were allowed to dry for 3–10 min. The meropenem disk from each TSB–meropenem suspension was placed on the previously inoculated MHA plate after removing any excess fluid. The MHA plates were inverted and incubated aerobically at 35 ± 2 °C for 18–24 h. After incubation, the zones of inhibition were measured.

For eCIM, an additional TSB tube was prepared for each bacterial isolate, to which 20 µL of 0.5 M EDTA was added. The same procedure as mCIM was then followed, with mCIM and eCIM tubes processed in parallel. Meropenem disks from both the mCIM and eCIM tubes were placed on the same MHA plate inoculated with the *E. coli* ATCC 25922 strain as described above.

In the case of mCIM, a zone diameter of 6–15 mm or the presence of pinpoint colonies within a 16–18 mm zone was interpreted as carbapenemase-positive, while a zone diameter of ≥19 mm was interpreted as carbapenemase-negative. The eCIM was interpreted only when the mCIM test was positive. An increase of ≥5 mm in zone diameter for the eCIM compared to the mCIM disk was interpreted as MBL-positive. An increase of ≤4 mm in zone diameter for the eCIM compared to the mCIM disk was interpreted as MBL-negative.

### 2.4. Genetic Identification and Characterization of AMR Determinants

The bacterial DNA of all CR-EC was extracted using a bacterial DNA extraction kit (FavorPrep™, Favorgen Biotech Corp., Ping Tung, Taiwan) according to the manufacturer’s instructions. DNA was stored at −70 °C for further analysis. The purity of DNA was determined via absorbance measured using Nano-Drop (Thermo Fisher Scientific, Cambridge, UK) at wavelengths of 260 and 280 nm. Species-specific primers for *E. coli* targeting the *uid*A gene were used (Supplementary Table S1) at 58 °C as the annealing temperature for 30 s with standard PCR conditions using conventional PCR [20].

All the CR-EC bacteria were screened for the extended-spectrum β-lactamase (ESBL)-encoding genes (*bla*_TEM_, *bla*_SHV_, and *bla*_CTX-M_ genes). The CR-EC bacteria that were found to be positive for *bla*_CTX-M_ were further screened for *bla*_CTX-M1_, _-M2_, _-M8_, _-M9_, _-M10_, _-M14_, and _-M15_. The isolates were also screened for the class B β-lactamases *bla*_NDM_, *bla*_IMP_, *bla*_VIM_, *bla*_SPM_, *bla*_KPC_, and *bla*_OXA-48_ using conventional PCR. *E. coli* ATCC 25922 was used as a negative control, and *bla*_NDM_-positive controls were acquired from a previous study (Mohsin Khurshid et al., 2017) [21]. *E. coli* NCTC-13476 for *bla*_IMP_, *Klebsiella pneumoniae* NCTC-13439 for *bla*_VIM_, *Klebsiella pneumoniae* ATCC-BAA1705 for *bla*_KPC,_ and *E. coli* ATCC-BAA2523 for *bla*_OXA-48_ were used as positive controls.

The screening for the 16S methylases, namely *arm*A, *npm*A, *rmt*A, *rmt*B, *rmt*C, *rmt*D, *rmt*E, *rmt*F, *rmt*G, and *rmt*H, was also performed. The primer sequences and relevant properties are mentioned in Supplementary Table S1. All of the primers used in this study were synthesized from Macrogen (Seoul, Republic of Korea). The PCR products were sequenced by means of the Sanger sequencing method from Macrogen (Seoul, Republic of Korea). The sequences were aligned and compared with the already available sequences in the NCBI database using the NCBI BLAST tool.

### 2.5. Molecular Characterization of bla_NDM_

The entire gene sequence of 984 bp was amplified using forward 5′-CACCTCATGTTTGAATTCGCC-3′ and reverse 5′-CTCTGTCACATCGAAATCGC-3′primers [22]. A 10 µL sample of the PCR reaction was prepared. PCR steps included an initial denaturation step of 2 min at 94 °C, followed by 35 cycles of amplification, each of which comprised 30 s at 94 °C, 40 s at 57 °C, and 45 s at 72 °C, with a final extension at 72 °C for 5 min. Post-PCR staining was performed to analyze PCR products after electrophoresis in 1% agarose gel in Tris base–acetic acid–EDTA (TAE) buffer at 95 V for 45 min. The *bla*_NDM_ forward and reverse primers were used to sequence the full-length *bla*_NDM_ gene. The sequencing facility of Macrogen (Seoul, Republic of Korea) was availed. The sequence data of both the strands of the PCR product were assembled using DNA Dragon Contig Sequence Assembly Software (version 1.5.0) SequentiX—Digital DNA Processing (Mecklenburg-Schwerin, Germany) (https://www.sequentix.de/software_dnadragon.php (accessed on 29 June 2023)). The consensus of a sequence originating from two different primer sets was developed for each component of bacteria.

### 2.6. MLST

MLST was performed for all CR-EC bacteria. Seven housekeeping genes (*adk*, *fum*C, *gyr*B, *icd*, *mdh*, *pur*A, and *rec*A) according to the conditions mentioned in the EnteroBase database were amplified [23]. The PCR products were purified using the GeneJET Gel Extraction Kit (Thermo Fisher Scientific, Waltham, MA, USA) and sequenced by Macrogen (Seoul, Republic of Korea). The sequences were analyzed and edited by DNA-Dragon software version 1.5.6 and were further aligned via the ClustalW algorithm using MEGA6 software version 6.0. Each of the gene loci was assigned an allelic number, and the STs were found following the respective allelic profiles for the isolates using the EnteroBase database.

### 2.7. Data Analysis

SPSS version 24 (IBM Corp. New York, NY, USA) was used for data analysis, and *p*-values < 0.05 were considered significant, using the linear-to-linear association test and chi-squared test.

## 3. Results

A total of 15,467 clinical samples were processed in the Microbiology Department at PIMS following standard guidelines. Positive cultures were identified in 6936 samples (44.84%), with 2712 (39.1%) classified as Enterobacterales. Susceptibility testing revealed that 944 isolates (34.81%) of Enterobacterales were non-susceptible to carbapenems. Among these, 155 isolates (16.42%) were identified as *E. coli* exhibiting MBL production, as confirmed by the eCIM test, and were selected for further investigation. Of these *E. coli* isolates, 20% (*n* = 31) were associated with community-acquired infections (CAIs), while 80% (*n* = 124) were associated with hospital-acquired infections (HAIs). Among CAIs, 45.16% (*n* = 14) were isolated from emergency departments and 54.84% (*n* = 17) were isolated from outpatient departments. For HAIs, 18.54% (*n* = 23) were isolated from intensive care units (ICUs; medical and surgical), 20.17% (*n* = 25) were isolated from medical wards, and 61.29% (*n* = 76) were isolated from surgical wards.

### 3.1. CR-EC Isolated from Different Sample Types and AMST of CR-EC Isolates

**Figure 1:**
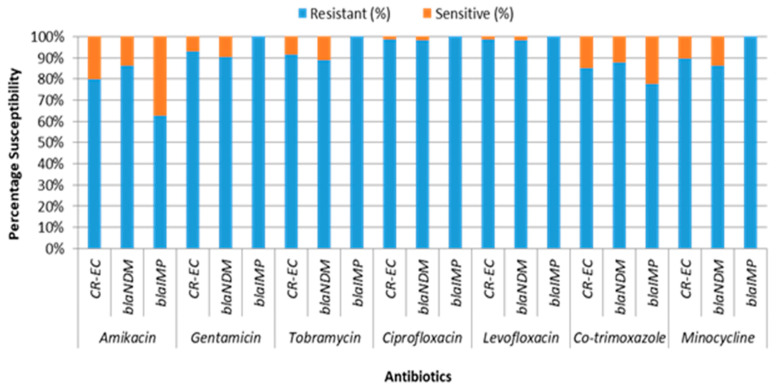
AMST of *bla*_NDM_- and *bla*_IMP_-harboring CR-EC isolates.

CR-EC was predominantly isolated from urine (42.58%) and aspirated pus (14.84%). It was also found in the blood (14.19%), tracheal secretions (11.61%), wound swabs (5.16%), and sputum (6.45%) (Table 1). Conversely, it was least frequently isolated from CVP tips (3.87%), bodily fluids (0.65%), and cerebrospinal fluid (0.65%). The CR-EC isolates exhibited exclusive resistance to ampicillin, amoxicillin/clavulanic acid, cefotaxime, ceftriaxone, cefepime, piperacillin/tazobactam, cefoperazone/tazobactam, imipenem, meropenem, and doripenem. All of these CR-EC isolates were MDR (resistant to one or more agent in more than three antimicrobial categories). The resistance rates against ciprofloxacin and levofloxacin were 98.71% each. The resistance rates to co-trimoxazole and minocycline were 85.16% and 89.68%, respectively. Among aminoglycosides, the resistance rates were 80% for amikacin, 91.61% for tobramycin, and 92.90% for gentamicin. In contrast, colistin, polymyxin B, and tigecycline showed complete sensitivity against all CR-EC isolates, as determined through MICs (Figure 1). The distribution of MICs (µg/mL) of CR-EC isolates is shown in Table 2. All CR-EC isolates were mCIM- and eCIM-test-positive, producing carbapenemases and MBLs.

### 3.2. Frequency, Co-Occurrence, and AMST of AMR-Determining Genes and bla_NDM_ Variants

The *bla*_VIM_, *bla*_GIM_, *bla*_SIM_, and *bla*_KPC_ genes were absent in CR-EC isolates. All the CR-EC isolates tested positive for either *bla*_NDM_ in 74.19% (115/155) or *bla*_IMP_ in 25.81% (40/155), with no isolates co-harboring both genes. All the eCIM-test-positive CR-EC were screened positive for MBL genes (*bla*_NDM_ or *bla*_IMP_). Among these, *bla*_OXA-48_ was also detected in 35.48% (55/155) of isolates. Among the ESBL genes, *bla*_SHV_ was found in 9.68% (15/155), *bla*_TEM_ in 73.55% (114/155), *bla*_CTX-M1_ in 18.06% (28/155), and *bla*_CTX-M15_ in 83.87% (130/155) (Table 2). Among the 16S methylases, *rmt*C, *rmt*D, *rmt*E, and *rmt*F were not detected. However, *arm*A was present in 40% (62/155) and *rmt*B was present in 65.16% (101/155) of CR-EC isolates. The *bla*_IMP_ and *bla*_OXA-48_ were recovered from urine in 35% and 34.55% of cases, respectively, and from aspirated pus in 22.5% and 20% of cases, respectively. The *bla*_SHV_, *bla*_TEM_, *bla*_CTX-M1_, and *bla*_CTX-M15_ were predominantly recovered from urine, with rates of 33.33%, 45.61%, 35.71%, and 43.85%, respectively. The *arm*A and *rmt*B were found in 41.94% and 44.55% of urine samples, respectively (Table 2).

The *bla*_NDM_-producing CR-EC isolates exhibited high resistance rates, with 98.26% showing resistance against both ciprofloxacin and levofloxacin. Resistance rates were also notable for co-trimoxazole (87.83%) and minocycline (86.09%). Among aminoglycosides, resistance rates were 86.09% for amikacin, 88.70% for tobramycin, and 90.43% for gentamicin (Figure 1). The MIC distribution of CR-EC isolates harboring *bla*_NDM_ and *bla*_IMP_ genes is illustrated in Table 1.

This study identified CR-EC isolates co-harboring various combinations of ESBL genes, MBL genes, and aminoglycoside resistance genes. Specifically, 19.34% and 16.13% of *bla*_OXA-48_ isolates co-harbored *bla*_NDM_ and *bla*_IMP_, respectively. The most frequent combinations of genes included *bla*_NDM_, *bla*_TEM_, *bla*_CTX-M15_, *arm*A, and *rmt*B (24.52%), followed by *bla*_NDM_, *bla*_TEM_, *bla*_CTX-M15_, and *rmt*B (23.23%) and *bla*_IMP_, *bla*_OXA-48_, *bla*_TEM_, *bla*_CTX-M1_, and *rmt*B (16.13%) (Table 3). The *arm*A and *rmt*B were co-harbored in 25.17% of CR-EC isolates. Among the 115 *bla*_NDM_-positive CR-EC isolates, variants included *bla*_NDM-1_ (66.96%), *bla*_NDM-5_ (12.17%), and *bla*_NDM-7_ (20.87%) (Table 2). The *bla*_NDM_ gene was predominantly recovered from urine (45.22%) and blood (15.65%). Specifically, 48.05% of *bla*_NDM-1_, 42.86% of *bla*_NDM-5_, and 37.5% of *bla*_NDM-7_ were isolated from urine, while 12.99% of *bla*_NDM-1_, 21.43% of *bla*_NDM-5_, and 20.83% of *bla*_NDM-7_ were isolated from blood (Table 2).

**Figure 2:**
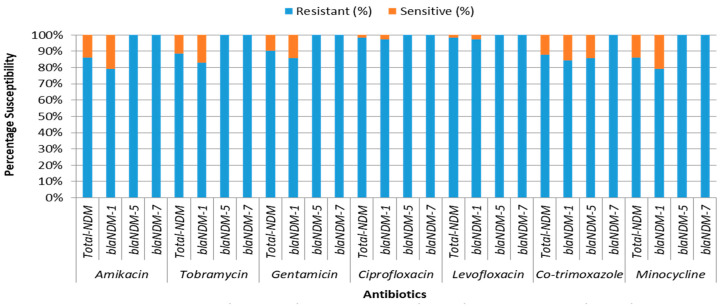
AMST of CR-EC isolates harboring different *bla*_NDM_ variants.

All *bla*_NDM_ variants exhibited exclusive resistance to all tested β-lactam drugs and carbapenems. The *bla*_NDM-7_- and *bla*_NDM-5_-producing bacteria showed additional exclusive resistance to ciprofloxacin, levofloxacin, amikacin, tobramycin, and gentamicin. The *bla*_NDM-7_-producing bacteria were also exclusively resistant to co-trimoxazole. The *bla*_NDM-1_ isolates exhibited 97.40% resistance to both ciprofloxacin and levofloxacin. The resistance rates for co-trimoxazole were 84.42% for *bla*_NDM-1_ and 85.71% for *bla*_NDM-5_. Additionally, *bla*_NDM-1_ and *bla*_NDM-5_ isolates showed resistance rates of 79.22% and 100% to minocycline, respectively. The resistance rates to amikacin, tobramycin, and gentamicin were 79.22%, 83.12%, and 85.71% for *bla*_NDM-1_-producing bacteria (Figure 2). The MIC distribution of *bla*_NDM_ variants is detailed in Table 1.

The *bla*_NDM-1_ was predominantly associated with the gene combination *bla*_NDM_, *bla*_TEM_, *bla*_CTX-M15_, and *rmt*B (23.23%), followed by *bla*_NDM_, *bla*_TEM_, *bla*_CTX-M15_, *arm*A, and *rmt*B (15.48%). The *bla*_NDM-5_ was exclusively associated with the gene combination *bla*_NDM_, *bla*_TEM_, *bla*_CTX-M15_, *arm*A, and *rmt*B (9.03%), and *bla*_NDM-7_ was predominantly associated with the gene combination *bla*_NDM_, *bla*_OXA-48_, *bla*_CTX-M15_, and *arm*A (14.84%) (Table 4).

### 3.3. MLST Sequence Typing and Association of AMR Genes with STs

The genetic relatedness of CR-EC bacteria was examined using MLST. The most prevalent STs were ST131 (*n* = 38, 24.52%) and ST2279 (*n* = 37, 23.87%), followed by ST3499 (*n* = 25, 16.13%) and ST8051 (*n* = 24, 15.48%) (Table 4). Other STs were ST8900 (9.68%), ST3329 (7.10%), ST88 (1.94%), and ST6293 (1.29%). Predominant clonal complexes (CCs) included ST131 complex (Cplx) (70.79%) and ST23 Cplx, (18.06%). Within ST131 Cplx, ST131 accounted for 34.55%, ST2279 for 33.64%, ST8051 for 21.82%, and ST3329 for 10%. ST23 Cplx exclusively comprised ST3499 (89.29%) and ST88 (10.71%). ST6293 and ST8900 were exclusively associated with ST38 and untypeable (NA) Cplx, respectively.

CR-EC bacteria harboring the *bla*_NDM_ gene predominantly belonged to ST131 Cplx (95.65%), encompassing ST131, ST2279, ST3329, and ST8051. Other CCs included ST23 Cplx (2.61%) containing ST88 and ST38 Cplx (1.74%) containing ST6293, which also harbored the *bla*_NDM_ gene. Bacteria harboring the *bla*_IMP_ gene were predominantly associated with ST23 Cplx (62.50%), particularly ST3499.

ST88, ST131, ST2279, ST3329, ST3499, ST8051, and ST8900 were predominantly isolated from urine samples, whereas ST6293 was recovered equally from blood (50%) and tracheal secretions (50%) (Table 5). The genetic diversity of *E. coli* strains harboring different AMR genes was assessed in terms of clonal lineage. ST131 acquired both *bla*_NDM-1_ (63.16%, *n* = 24) and *bla*_NDM-5_ (36.84%, *n* = 14) variants. ST8051 was exclusively associated with *bla*_NDM-7_. ST88, ST2279, ST3329, and ST6293 were exclusively associated with the *bla*_NDM-1_ only (Table 6).

It was observed that different AMR genes were harbored by different STs. Among *E. coli* strains, MBL genes were found in various STs: *bla*_NDM-1_ in ST88 (100%, 3/3), ST131 (63.16%, 24/38), and ST2279 (100%, 37/37); *bla*_NDM-5_ in ST131 (36.84%, 14/38); *bla*_NDM-1_ and *bla*_OXA-48_ in ST3329 (100%, 11/11 and 54.55%, 6/11, respectively); *bla*_IMP_ and *bla*_OXA-48_ in ST3499 (100%, 25/25); *bla*_NDM-1_ in ST6293 (100%, 2/2); *bla*_NDM-7_ and *bla*_OXA-48_ in ST8051 (100%, 24/24); and *bla*_IMP_ in ST8900 (100%, 15/15) (Table 6).

## 4. Discussion

The findings of this study reveal a significant prevalence of carbapenem-resistant Enterobacterales (CRE) within a large tertiary care hospital. Among the 15,467 clinical samples processed, a substantial proportion (44.84%) yielded positive cultures, with 39.1% identified as Enterobacterales. Notably, carbapenem resistance was observed in 34.81% of these isolates, with *E. coli* constituting 16.42% of the CRE population. Most of these CR-EC isolates were involved in hospital-acquired infections (HAIs), representing 80% of cases, particularly prevalent in ICUs and medical wards. Molecular analysis revealed the predominance of the *bla*_NDM_ gene in 74.19% of CR-EC isolates, followed by *bla*_IMP_ in 25.81%. Additionally, the presence of blaOXA-48, detected in 35.48% of isolates, and the detection of ESBL genes, particularly *bla*_TEM_ and *bla*_CTX-M15_, along with 16S methylases, further emphasize the multifaceted nature of antibiotic resistance in these clinical settings. In this study, we present the molecular epidemiology of CR-EC isolated from the largest tertiary care hospital in the capital city of Pakistan. Limited data from federal institutions in Pakistan are available, with only one previous study investigating the epidemiology of MBL-producing *E. coli* using MLST [16].

MBLs, particularly the *bla*_NDM_ gene, represent emerging β-lactamases that significantly contribute to the development of carbapenem-resistant phenotypes in Gram-negative bacteria. This gene has attracted global attention and concern due to its rapid dissemination and capacity to confer resistance to last-line antibiotics [24]. Developing countries, such as Pakistan, face substantial challenges in managing infections caused by *bla*_NDM_-producing bacteria, exacerbated by limited resources, fragile healthcare systems, and higher rates of infectious diseases [25]. Conducting extensive and comprehensive studies on the molecular epidemiology of *bla*_NDM_ variants among Gram-negative bacteria is imperative to gain a precise understanding of their prevalence, spread, and genetic characteristics [26]. Globally, the incidence of isolates carrying the *bla*_NDM_ gene is increasing. Reports confirm that *bla*_NDM_-carrying isolates are present in various regions worldwide [7]. These studies offer valuable insights into the dynamics of *bla*_NDM_ transmission, the genetic diversity of its variants, and their associations with specific bacterial species or clones [27]. Furthermore, such research can provide critical data to enhance antimicrobial stewardship policies in endemic regions [28]. In Asia, countries like India, Pakistan, and China report a high prevalence of *bla*_NDM_-carrying isolates, establishing these regions as major reservoirs and hotspots for *bla*_NDM_ dissemination, significantly contributing to its global spread [29,30].

In our study, the prevalence of CR-EC isolates was 15.6%. We observed a high frequency of the *bla*_NDM_ gene at 74.19% (*n* = 115), significantly higher than the *bla*_IMP_ gene at 25.81% (*n* = 40), contributing to carbapenem resistance. Previous studies from Pakistan reported varying levels of carbapenem resistance in *E. coli*, ranging from 7.0% to 37.97% [31,32,33,34]. In a recent Pakistani study, 22.02% of *E. coli* isolates were carbapenemase producers, with 66.67% of these being MBL producers. The incidence of *bla*_NDM_ was reported at 35.83%, followed by *bla*_KPC-2_ (26.67%), *bla*_VIM_ (25%), *bla*_IMP-1_ (20.83%), and *bla*_OXA-48_ (8.3%), among these MBL producers [16]. Globally, varying rates of CR-EC dissemination have been documented, such as 0.02% in the Netherlands [35], 3.4% with 85.19% *bla*_NDM_ producers in China [36], 4.27% with 80% *bla*_NDM_ producers in Nepal [7], 14% with 76.19% *bla*_NDM_ in India [37], 27.1% in Egypt [38], 59% in Vietnam [39], and 63.9% in Greece [40]. The prevalence of CR-EC in our study aligns with findings from India, while the prevalence of *bla*_NDM_ is comparable to rates observed in China, Nepal, and India.

Various studies from Pakistan have identified several variants of *bla*_NDM_, including *bla*_NDM-1_, *bla*_NDM-4_, *bla*_NDM-5_, and *bla*_NDM-7_, among CR-EC MBL producers. The prevalence of *bla*_NDM-4_ (0 to 11.1%) and *bla*_NDM-7_ (0 to 12.3%) in Pakistan is notably low; whereas, *bla*_NDM-1_ (33.3% to 52.6%) has been reported as the predominant genotype compared to *bla*_NDM-5_ (0 to 50.5%) in previous studies [28,33,41,42,43]. In our current study, *bla*_NDM-1_ was the most prevalent (66.96%), followed by *bla*_NDM-7_ (20.87%) and *bla*_NDM-5_ (12.17%). *bla*_NDM-4_ was not detected in our study.

Tigecycline and colistin are often considered last-resort treatments, either alone or in combination with other antibiotics, for severe infections. Colistin, particularly effective for complicated urinary tract infections (UTIs), is used either alone or in combination with aminoglycosides when physicians are left with no other options. In our study, all CR-EC isolates were exclusively susceptible to colistin, consistent with findings from other studies [7,28,44]. Contradicting our findings, colistin resistance mediated by the *mcr*-1 gene has been reported in CR-EC isolates from Pakistan and globally [16,45]. These findings may correlate with the injudicious use of these antibiotics and the type of circulating bacterial clones in particular hospitals. The distribution of MICs can vary geographically and over time, reflecting local resistance patterns and antibiotic usage practices. Regular surveillance and susceptibility testing are essential for effective antimicrobial stewardship.

Containment strategies in healthcare settings are pivotal for preventing and slowing the spread of CR-EC strains. These strategies typically encompass comprehensive measures such as antimicrobial stewardship, infection prevention and control (IPC) protocols, surveillance, monitoring, and promoting rational antibiotic use. In our institution, regular monitoring of bacterial resistance is conducted, and the antibiotic administrative rationale is periodically reviewed, but as our institution is a single tertiary care hospital with a high number of referrals from other cities and provinces, its patient load is very high. Therefore, it is difficult to adhere to IPC practices, which are very poor in our institution. The government needs to develop more health facilities to overcome the basic demand driven by the increasing population and urban influx. By expanding and implementing such plans, healthcare facilities can effectively mitigate the dissemination of resistant bacteria and preserve the efficacy of antimicrobial therapies.

In our study, 35.48% of CR-EC isolates harbored *bla*_OXA-48_. Among these, 19.34% co-harbored *bla*_OXA-48_ with *bla*_IMP_ and 16.13% with *bla*_NDM_. A previous study conducted in Islamabad reported *bla*_NDM-1_, *bla*_IMP_, and *bla*_OXA-48_ prevalence at 35.83%, 20.83%, and 8.3%, respectively. Additionally, *bla*_KPC-2_ (26.67%) and *bla*_VIM-1_ (25%) genes were also documented in this previous study [16]. Another study from Pakistan found that 21.1% of *bla*_OXA-48_-positive isolates co-occurred with *bla*_NDM_ (*n* = 15) and *bla*_IMP_ (*n* = 8), with no *bla*_NDM_ and *bla*_IMP_ co-harboring in CR-EC isolates [28]. The comparably high prevalence of *bla*_NDM_, *bla*_IMP_, and *bla*_OXA-48_, the absence of *bla*_KPC_ and *bla*_VIM_, and the relatively high co-occurrence of *bla*_OXA-48_ with *bla*_NDM_ rather than with *bla*_IMP_ in our study underscore the importance of interpreting these differences in light of geographical location, sample size, source, and type.

In our study, *bla*_CTX-M_ was exclusively detected in all CR-EC isolates. Further testing revealed that *bla*_CTX-M15_ was the most prevalent, at 83.87%, followed by *bla*_TEM_ (73.55%) and *bla*_SHV_ (9.68%). All isolates also co-harbored both MBL and ESBL genes. In another study from Pakistan, *bla*_CTX-M_ (71.6%) was similarly the predominant genotype, followed by *bla*_TEM_ (59.6%), with no detection of *bla*_SHV_ and all isolates co-harboring MBL and ESBL genes [28]. Another study from Pakistan reported a 51.07% co-existence of MBL and ESBL genes [16]. Recent data on 16S rRNA methylase genes in Pakistan are limited. In our study, the frequencies of *rmt*B and *arm*A genes were 65.16% and 40%, respectively. A previous study reported frequencies of 63.3% for *rmt*B and 50.5% for *arm*A in CR-EC isolates [28]. In our recent study, 49.05% and 39.99% of *bla*_NDM_-producing CR-EC isolates co-harbored *rmt*B and *arm*A 16S rRNA methylase genes, respectively (Table 3). Only 16.13% of *rmt*B co-harbored with *bla*_IMP_, and *arm*A was not found in combination with *bla*_IMP_. Previous studies in Pakistan indicated a low association of 16S rRNA methylase with ESBLs and *bla*_NDM_-producing *E. coli* [46,47,48]. Isolates co-harboring ESBLs, MBLs, and 16S rRNA methylases are increasingly challenging for clinicians to treat. In our study, the most prevalent combination was 25.52% with *bla*_NDM_, *bla*_TEM_, *bla*_CTX-M_, *bla*_CTX-M15_, *arm*A, and *rmt*B genes, followed by 23.23% with *bla*_NDM_, *bla*_TEM_, *bla*_CTX-M_, *bla*_CTX-M15_, and *rmt*B genes.

The emergence and spread of various STs with carbapenem resistance, facilitated by the acquisition of relevant plasmids in healthcare environments, significantly contributes to the challenge of antimicrobial resistance in the region. In our study, we identified different CR-EC STs harboring *bla*_NDM_, including ST131, which differs from findings in previous studies. Recent research conducted in Rawalpindi detected ST167, ST405, ST410, and ST617 [42], while studies carried out in Lahore reported ST101, ST156, ST405, and ST648 [35,43]. ST10 (9.7–16.0%), ST69 (7.4%), ST101 (9.7–19.7%), ST131 (29.2–21.2%), ST405 (34.5–44.4%), and ST648 (7%) were also identified in Lahore in various studies, harboring different combinations of *bla*_NDM-7_, *bla*_NDM-1_, *bla*_OXA-48_, and ESBL genes [31,33]. In a study from the Southern Punjab province of Pakistan, ST131 was the most prevalent clone (37.8%), similar to our findings (24.52%), and harbored *bla*_NDM-1_ and *bla*_NDM-5_ [28]. Despite the extensive study of the epidemiology of the ST131 clone, significant gaps remain in understanding its transmission mechanisms, reservoirs, and associated risk factors. Risk factors for acquiring ST131 isolates may include prolonged residence in healthcare facilities or nursing homes, older age, diabetes mellitus, surgical interventions, cancer, exposure to antibiotics, lack of prior antibiotic treatment, and use of proton pump inhibitors [49].

ST131 Cplx and ST23 Cplx are widely recognized clonal complexes of *E. coli* known for harboring virulence factors and AMR genes, which contribute to MDR infections globally. These CCs frequently carry ESBL genes, and MBL genes have also been associated with ST131 Cplx and ST23 Cplx [5]. The ST131 clone of *E. coli*, in particular, demonstrates a heightened ability to acquire resistance determinants under selective pressure, enhancing its success as an MDR pathogen [50]. In our study, ST131 Cplx was the predominant CC (70.97%), encompassing ST131 (34.55%), ST3329 (10%), ST2279 (33.64%), and ST8051 (21.82%). These complexes carried *bla*_NDM-1_, *bla*_NDM-5_, or *bla*_NDM-7_, with ST131 isolates concurrently harboring both *bla*_NDM-1_ and *bla*_NDM-5_. ST131 *E. coli* strains carrying ESBL and MBL genes have also been isolated from animals and the environment, underscoring the importance of the “One Health” concept to understand the transmission dynamics of such clones across poultry, livestock, and clinical settings [6].

In our present study, carbapenem resistance mechanisms beyond carbapenemase production were not explored, and plasmid replicon typing was not performed. However, comprehensive, multicenter studies are crucial to further characterize CR-EC and enhance our understanding of the molecular epidemiology of prevalent clones in the region. Such studies are essential for identifying additional resistance mechanisms and elucidating the role of plasmids in disseminating resistance genes among CR-EC strains.

## 5. Conclusions

This study highlights the alarming prevalence of carbapenem-resistant *E. coli* within a major tertiary care hospital in Pakistan. The widespread presence of the *bla*_NDM_ gene, along with other resistance genes such as *bla*_IMP_ and *bla*_OXA-48_, reflects a growing public health concern that aligns with global trends, particularly in regions like Asia. The detection of various sequence types, particularly ST131, which harbors multiple resistance genes, highlights the complex epidemiology of CRE in healthcare settings and the need for targeted infection control measures. This study also emphasizes the importance of continuous surveillance and molecular epidemiology to track the spread of these resistance determinants. Future studies should explore additional resistance mechanisms and the role of plasmids in the dissemination of resistance genes to develop more effective containment strategies.

## Figures and Tables

**Table 1:** Distribution of CR-EC, AMR genes, and *bla*_NDM_ variants among different specimens.

Specimens	CR-EC	*bla*_NDM-1_	*bla*_NDM-5_	*bla*_NDM-7_	*bla*_IMP_	*bla*_OXA-48_	*bla*_SHV_	*bla*_TEM_	*bla*_CTX-M1_	*bla*_CTX-M15_	*arm*A	*rmt*B
Blood	22 (14.19%)	10 (12.99%)	3 (21.43%)	5 (20.83%)	4 (10%)	8 (14.55%)	2 (13.33%)	14 (12.28%)	3 (10.71%)	20 (15.38%)	10 (16.13%)	13 (12.87%)
CSF	1 (0.65%)	1 (1.30%)	0	0	0	0	0	1 (0.88%)	0	1 (0.77%)	1 (1.61%)	1 (0.99%)
Body Fluid	1 (0.65%)	1 (1.30%)	0	0	0	0	0	1 (0.88%)	0	1 (0.77%)	1 (1.61%)	1 (0.99%)
Aspirated Pus	23 (14.84%)	10 (12.99%)	1 (7.14%)	3 (12.50%)	9 (22.50%)	11 (20%)	3 (20%)	17 (14.91%)	6 (21.43%)	17 (13.08%)	7 (11.29%)	14 (13.86%)
Sputum	10 (6.45%)	4 (5.19%)	1 (7.14%)	1 (4.17%)	4 (10%)	5 (9.09%)	1 (6.67%)	8 (7.02%)	3 (10.71%)	7 (5.38%)	5 (8.06%)	7 (6.93%)
Tip	6 (3.87%)	1 (1.30%)	1 (7.14%)	1 (4.17%)	3 (7.50%)	3 (5.45%)	1 (6.67%)	4 (3.51%)	2 (7.14%)	4 (3.08%)	2 (3.23%)	4 (3.96%)
Tracheal Secretion	18 (11.61%)	9 (11.69%)	2 (14.29%)	3 (12.50%)	4 (10%)	7 (12.73%)	1 (6.67%)	13 (11.40%)	4 (14.29%)	15 (11.54%)	7 (11.29%)	12 (11.88%)
Urine	66 (42.58%)	37 (48.05)	6 (42.86%)	9 (37.50%)	14 (35%)	19 (34.55%)	5 (33.33%)	52 (45.61%)	10 (35.71%)	57 (43.85%)	26 (41.94%)	45 (44.55%)
Wound Swab	8 (5.16%)	4 (5.19%)	0	2 (8.33%)	2 (5%)	2 (3.64%)	2 (13.33%)	4 (3.51%)	0	8 (6.15%)	3 (4.84%)	4 (3.96%)
Total	N = 155	N = 77 (66.96%)	N = 14 (12.17%)	N = 24 (20.87%)	N = 40 (25.81%)	N = 55 (35.48%)	N = 15 (9.68%)	N = 114 (73.55%)	N = 28 (18.06%)	N = 130 (83.87%)	N = 62 (40%)	N = 101 (65.16%)

**Table 2:** MIC distribution of *bla*_NDM_- and *bla*_IMP_-harboring CR-EC isolates.

Antimicrobial Agent	Range (µg/mL)	Resistance Trait	Number of Isolates with MIC of (µg/mL)													MIC_50_	MIC_90_
			<0.06	0.125	0.25	0.5	1	2	4	8	16	32	64	128	≥256		
Cefepime	0.5–512	Total	-	-	-	-	-	-	-	-	-	-	-	2	153	≥256	≥256
		*bla*_NDM_	-	-	-	-	-	-	-	-	-	-	-	2	110	≥256	≥256
		*bla*_IMP_	-	-	-	-	-	-	-	-	-	-	-	-	40	≥256	≥256
Cefotaxime	0.5–512	Total	-	-	-	-	-	-	-	-	-	-	-	-	155	≥256	≥256
		*bla*_NDM_	-	-	-	-	-	-	-	-	-	-	-	-	115	≥256	≥256
		*bla*_IMP_	-	-	-	-	-	-	-	-	-	-	-	-	40	≥256	≥256
Ceftriaxone	0.5–512	Total	-	-	-	-	-	-	-	-	-	-	-	-	155	≥256	≥256
		*bla*_NDM_	-	-	-	-	-	-	-	-	-	-	-	-	115	≥256	≥256
		*bla*_IMP_	-	-	-	-	-	-	-	-	-	-	-	-	40	≥256	≥256
Imipenem	0.5–512	Total	-	-	-	-	-	-	45	76	24	10	-	-	-	8	16
		*bla*_NDM_	-	-	-	-	-	-	24	57	24	10	-	-	-	8	16
		*bla*_IMP_	-	-	-	-	-	-	21	19	-	-	-	-	-	4	8
Meropenem	0.5–512	Total	-	-	-	-	-	-	19	61	50	20	5	-	-	8	32
		*bla*_NDM_	-	-	-	-	-	-	8	45	37	20	5	-	-	16	32
		*bla*_IMP_	-	-	-	-	-	-	11	16	13	-	-	-	-	8	16
Ciprofloxacin	0.5–512	Total	-	-	-	2	-	-	-	-	-	39	55	34	25	64	≥256
		*bla*_NDM_	-	-	-	2	-	-	-	-	-	20	45	30	18	64	≥256
		*bla*_IMP_	-	-	-	-	-	-	-	-	-	19	10	4	7	64	≥256
Amikacin	0.5–512	Total	-	-	-	9	19	3	-	-	-	-	10	12	102	≥256	≥256
		*bla*_NDM_	-	-	-	3	10	3	-	-	-	-	10	12	77	≥256	≥256
		*bla*_IMP_	-	-	-	6	9	-	-	-	-	-	-	-	25	≥256	≥256
Gentamicin	0.5–512	Total	-	-	-	4	7	-	-	-	8	19	14	3	100	≥256	≥256
		*bla*_NDM_	-	-	-	4	7	-	-	-	3	9	14	3	75	≥256	≥256
		*bla*_IMP_	-	-	-	-	-	-	-	-	5	10	-	-	25	≥256	≥256
Tobramycin	0.5–512	Total	-	-	-	5	8	-	-	2	13	8	18	13	88	≥256	≥256
		*bla*_NDM_	-	-	-	5	8	-	-	1	2	5	18	13	63	≥256	≥256
		*bla*_IMP_	-	-	-	-	-	-	-	1	11	3	-	-	25	≥256	≥256
Colistin	0.06–32	Total	-	9	10	65	70	1	-	-	-	-	-	-	-	0.5	1
		*bla*_NDM_	-	9	5	40	60	1	-	-	-	-	-	-	-	1	1
		*bla*_IMP_	-	-	5	25	10	-	-	-	-	-	-	-	-	0.5	1
Polymyxin B	0.06–32	Total	-	23	20	55	56	1	-	-	-	-	-	-	-	0.5	1
		*bla*_NDM_	-	19	7	39	49	1	-	-	-	-	-	-	-	0.5	1
		*bla*_IMP_	-	4	13	16	7	-	-	-	-	-	-	-	-	0.5	1
Tigecycline	0.06–32	Total	-	-	22	57	39	37	-	-	-	-	-	-	-	0.5	2
		*bla*_NDM_	-	-	16	38	26	35	-	-	-	-	-	-	-	1	2
		*bla*_IMP_	-	-	6	19	13	2	-	-	-	-	-	-	-	0.5	1

**Table 3:** Co-occurrence of AMR genes among CR-EC isolates.

MBL-Encoding Genes			ESBL-Encoding Genes				Aminoglycoside Resistance Conferring Genes		No. (%)
*bla*_NDM_	-	-	-	*bla*_TEM_	-	*bla*_CTX-M15_	*arm*A	*rmt*B	38 (24.52)
*bla*_NDM_	-	-	-	*bla*_TEM_	-	*bla*_CTX-M15_	-	*rmt*B	36 (23.23)
*bla*_NDM_	-	*bla*_OXA-48_	-	-	-	*bla*_CTX-M15_	*arm*A	-	23 (14.82)
*bla*_NDM_	-	-	-	*bla*_TEM_	-	*bla*_CTX-M15_	-	-	8 (5.16)
*bla*_NDM_	-	*bla*_OXA-48_	-	*bla*_TEM_	-	*bla*_CTX-M15_	-	-	6 (3.87)
*bla*_NDM_	-	-	-	-	*bla*_CTX-M1_	*bla*_CTX-M15_	-	-	2 (1.29)
*bla*_NDM_	-	*bla*_OXA-48_	-	-	-	*bla*_CTX-M15_	*arm*A	*rmt*B	1 (0.65)
*bla*_NDM_	-	-	-	*bla*_TEM_	*bla*_CTX-M1_	*bla*_CTX-M15_	-	*rmt*B	1 (0.65)
-	*bla*_IMP_	*bla*_OXA-48_	-	*bla*_TEM_	*bla*_CTX-M1_	-	-	*rmt*B	25 (16.13)
-	*bla*_IMP_	-	*bla*_SHV_	-	-	*bla*_CTX-M15_	-	-	15 (9.68)

**Table 4:** *E. coli* STs harboring various AMR gene combinations.

STs N (%)								Total *n* (%)	Gene Combination
ST88	ST131	ST2279	ST3329	ST3499	ST6293	ST8051	ST8900		
-	24 (63.16%)	-	-	-	-	-	-	24 (15.48%)	*bla*_NDM-1_, *bla*_TEM_, *bla*_CTX-M15_, *arm*A, *rmt*B
-	-	1 (2.70%)	-	-	-	-	-	1 (0.65%)	*bla*_NDM-1_, *bla*_TEM_, *bla*_CTX-M1_, *bla*_CTX-M15_, *rmt*B
-	-	-	6 (54.55%)	-	-	-	-	6 (3.87%)	*bla*_NDM-1_, *bla*_OXA-48_, *bla*_TEM_, *bla*_CTX-M15_
-	-	36 (97.30%)	-	-	-		-	36 (23.23%)	*bla*_NDM-1_, *bla*_TEM_, *bla*_CTX-M15_, *rmt*B
3 (100%)	-	-	5 (45.45%)	-	-	-	-	8 (5.16%)	*bla*_NDM-1_, *bla*_TEM_, *bla*_CTX-M15_
-	-	-		-	2 (100%)	-	-	2 (1.29%)	*bla*_NDM-1_, *bla*_CTX-M1_, *bla*_CTX-M15_
-	14 (36.84%)	-	-	-	-	-	-	14 (9.03%)	*bla*_NDM-5_, *bla*_TEM_, *bla*_CTX-M15_, *arm*A, *rmt*B
-	-	-	-	-	-	1 (4.17%)	-	1 (0.65%)	*bla*_NDM-7_, *bla*_OXA-48_, *bla*_CTX-M15_, *arm*A, *rmt*B
-	-	-	-	-		23 (95.83%)	-	23 (14.84%)	*bla*N_DM-7_, *bla*_OXA-48_, *bla*_CTX-M15_, *arm*A
-	-	-	-	-	-	-	15 (100%)	15 (9.68%)	*bla*_IMP_, *bla*_SHV_, *bla*_CTX-M15_
-	-	-	-	25 (100%)	-	-	-	25 (16.13%)	*bla*_IMP_, *bla*_OXA-48_, *bla*_TEM_, *bla*_CTX-M1_, *rmt*B

**Table 5:** Specimen-wise distribution of STs.

STs	N (%)	Blood	CSF	Fluid	Pus	Sputum	Tip	Tracheal Secretion	Urine	Wound Swab
ST88	3 (1.94%)	1 (4.55%)	-	-	-	-	-	-	2 (3.03%)	-
ST131	38 (24.52%)	5 (22.73%)	1 (100%)	1 (100%)	4 (17.39%)	4 (40%)	1 (16.67%)	4 (22.22%)	17 (25.76%)	1 (12.50%)
ST2279	37 (23.87%)	5 (22.73%)	-	-	4 (17.39%)	0	1 (16.67%)	5 (27.78%)	19 (28.79%)	3 (37.50%)
ST3329	11 (7.10%)	1 (4.55%)	-	-	3 (13.04%)	1 (10%)	0	1 (5.56%)	5 (7.58%)	0
ST3499	25 (16.13%)	2 (9.09%)	-	-	6 (26.09%)	3 (30%)	2 (33.33%)	3 (16.67%)	9 (13.64%)	0
ST6293	2 (1.29%)	1 (4.55%)	-	-	0	0	0	1 (5.56%)	0	0
ST8051	24 (15.48%)	5 (22.73%)	-	-	3 (13.04%)	1 (10%)	1 (16.67%)	3 (16.67%)	9 (13.64%)	2 (25%)
ST8900	15 (9.68%)	2 (9.09%)	-	-	3 (20.00)	1 (10%)	1 (16.67%)	1 (5.56%)	5 (7.58%)	2 (25%)

**Table 6:** Distribution of AMR genes among different STs.

STs	Isolates *n* (%)	MBL-Encoding Genes					ESBL- Encoding Genes				Aminoglycoside Resistance Conferring Genes	
		*bla*_NDM-1_	*bla*_NDM-5_	*bla*_NDM-7_	*bla*_IMP_	*bla*_OXA-48_	*bla*_SHV_	*bla*_TEM_	*bla*_CTX-M1_	*bla*_CTX-M15_	*arm*A	*rmt*B
88	3 (1.94%)	3 (3.90%)	-	-	-	-	-	3 (2.63%	-	3 (2.31%)	-	-
131	38 (24.52%)	24 (31.17%)	14 (100%)	-	-	-	-	38 (33.33%)	-	38 (29.23%)	38 (61.29%)	38 (37.62%)
2279	37 (23.87%)	37 (48.05%)	-	-	-	-	-	37 (32.46%)	1 (3.57%)	37 (28.46%)	-	37 (36.63%)
3329	11 (7.10%)	11 (14.29%)	-	-	-	6 (10.91%)	-	11 (9.65%)	-	11 (8.46%)	-	-
3499	25 (16.13%)	-	-	-	25 (62.50%)	25 (45.45%)	-	25 (21.93%)	25 (89.29%)	0	-	25 (24.75%)
6293	2 (1.29%)	2 (2.60%)	-	-	0	0	-	-	2 (7.14%)	2 (1.54%)	-	-
8051	24 (15.48%)	-	-	24 (100%)	0	24 (43.64%)	-	-	-	24 (18.46%)	24 (38.71%)	1 (0.99%)
8900	15 (9.68%)	-	-	0	15 (37.50%)	0	15 (100%)	-	-	15 (11.54%)	-	-

## Data Availability

The data presented in this study are available in this article.

## Supplementary Materials

The following supporting information can be downloaded at: https://www.mdpi.com/article/10.3390/medicina60091528/s1, Table S1: Primers used in this study.
