# Supplementary material for: Predominance of blaNDM- and blaIMP-Harboring Escherichia coli Belonging to Clonal Complexes 131 and 23 in a Major University Hospital
Source: Medicina (Kaunas). 2024 Sep 19;60(9):1528. doi: 10.3390/medicina60091528 (PMC11434522; doi:10.3390/medicina60091528)
Supplement: Supplementary file 1 [file medicina-60-01528-s001.zip › medicina-3170013-supplementary.pdf]

Table S1. Primers used in study for the amplification of antimicrobial resistance genes.

S.#	Gene Name	Primer Name	Primer Sequence F 5'-3' R 3'-5'	Size of Product (bp)	Annealing Temperature (°C)
**a. Aminoglycoside resistance conferring genes**					
1	armA	armA-F<br>armA-R	ATTCTGCCTATCCTAATTGG<br>ACCTATACTTTATCGTCGTC	315	50
2	rmtA	rmtA-F<br>rmtA-R	CTAGCGTCCATCCTTTCCTC<br>TTTGCTTCCATGCCCTTGCC	371	50
3	rmtB	rmtB-F<br>rmtB-R	ATGAACATCAACGATGCCCT<br>CCTTCTGATTGGCTTATCCA	769	50
4	rmtC	rmtC-F<br>rmtC-R	CGAAGAAGTAACAGCCAAAG<br>ATCCCAACATCTCTCCCACT	711	50
5	rmtD	rmtD-F<br>rmtD-R	CGGCACGCGATTGGGAAGC<br>CGGAAACGATGCGACGAT	401	55
6	rmtE	rmtE-F<br>rmtE-R	ATGAATATTGATGAAATGGTTGC<br>TGATTGATTTCCTCCGTTTTTG	823	50
7	rmtF	rmtF-F<br>rmtF-R	GCGATACAGAAAACCGAAGG<br>ACCAGTCGGCATAGTGCTTT	589	52
8	rmtG	rmtG-F<br>rmtG-R	AAATACCGCGATGTGTGTCC<br>ACACGGCATCTGTTTCTTCC	250	55
9	rmtH	rmtH-F<br>rmtH-R	AATGACCATTGAACAGGCAGC<br>TCAAGCTGGGTTTGGCTGGA	760	55
**b. Carbapenemase producing genes**					
10	IMP	IMP-F<br>IMP-R	GGAATAGAGTGGCTTAAYTC<br>TCGGTTTAAYAAAACAACCACC	232	50
11	NDM	NDM-F<br>NDM-R	CACCTCATGTTTGAATTCGCC<br>CTCTGTCACATCGAAATCGC	984	52
12	VIM	VIM-F<br>VIM-R	GATGGTGTTTGGTCGCATA<br>CGAATGCGCAGCACCAG	390	50
13	GIM	GIM-F<br>GIM-R	TCGACACACCTTGGTCTGAA<br>AACTTCCAACTTTGCCATGC	477	52
14	SIM	SIM-F<br>SIM-R	TACAAGGGATTCGGCATCG<br>TAATGGCCTGTTCCCATGTG	551	56
15	KPC	KPC-F<br>KPC-R	CGTCTAGTTCTGCTGTCTTG<br>CTTGTCATCCTTGTTAGGCG	798	50
16	OXA-48	OXA48-F<br>OXA48-R	TTGGTGGCATCGATTATCGG<br>GAGCACTTCTTTTGTGATGGC	744	52
**c. ESBL producing genes**					
17	SHV	SHV-F<br>SHV-R	ATGCGTTATATTCGCCTGTG<br>AGATAAATCACCACAATGCGC	896	56
18	TEM	TEM-F<br>TEM-R	TCAACATTTCCGTGTCG<br>CTGACAGTTACCAATGCTTA	860	56
19	CTX-M	CTXM-F<br>CTXM-R	ATGTGCAGYACCAGTAARGT<br>TGGGTRAARTARGTSACCAGA	593	52
20	CTX-M1	CTXM1-F<br>CTXM1-R	CCGTTTCCGCTATTACAAACCGTTG<br>GGCCCATGGTTAAAAAATCACTGC	944	56
21	CTX-M15	CTXM15-F<br>CTXM15-R	CACACGTGGAATTTAGGGACT<br>GCCGTCTAAGGCGATAAACA	996	55
